# Sick Leave in Patients with Inflammatory Bowel Disease Before and After Diagnosis: A Retrospective Matched Cohort Study in Germany

**DOI:** 10.3390/epidemiologia7050126

**Published:** 2026-09-10

**Authors:** Annika Fernandez Milano, Sarah Krieg, Karel Kostev

**Affiliations:** 1 Epidemiology, IQVIA, 60549 Frankfurt am Main, Germany; 2 Department of Inclusive Medicine, University Clinic for People with Neurodevelopment Disorders, Mara Hospital, Medical School and University Medical Center OWL, Bielefeld University, 33617 Bielefeld, Germany; 3 Philipps-University, University Clinic, 35043 Marburg, Germany

**Keywords:** inflammatory bowel disease, ulcerative colitis, Crohn’s disease, sick leave, sickness absence, real-world data

## Abstract

Background/Objectives: Inflammatory bowel disease (IBD) can affect work participation. Evidence on sick leave among IBD patients in Germany is limited. We compared the occurrence and duration of certified sick leave between IBD patients and non-IBD controls. Methods: Practice data from the Disease Analyzer database in Germany were used. Newly diagnosed IBD patients were matched to non-IBD controls using propensity scores. Sick leave occurrence and duration were evaluated in the year before and two years after diagnosis, using logistic and zero-inflated negative binomial regression, respectively. Stratified analyses explored variations by IBD subtype, sex, and age group. Results: Sick leave occurred more frequently in IBD patients than controls pre-index (45.7% vs. 42.2%) and during the second year post-index (49.6% vs. 42.5%), consistent with higher odds in these periods (odds ratio (OR) 1.17, 95% confidence interval (CI) 1.11–1.22 and OR 1.35, 95% CI 1.29–1.42, respectively) but not during the first year post-index, when proportions were highest in both cohorts (56.2% vs. 56.4%). While median sick leave durations were low in both cohorts (0–2 days), mean durations were higher among IBD patients. In the second year post-index, IBD patients had 1.59 times (95% CI 1.50–1.69) more sick leave days than controls. Stratified analyses suggested higher sick leave estimates in ulcerative colitis than Crohn’s disease, more frequent sick leave in younger patients, and longer mean sick leave durations in older patients. Conclusions: IBD is associated with a greater certified sick leave burden before and after diagnosis, although absolute differences compared with controls were modest.

## 1. Introduction

Inflammatory bowel diseases (IBD), comprising Crohn’s disease (CD) and ulcerative colitis (UC), affect approximately 4.9 million individuals worldwide [1] and up to 470,000 in Germany [2,3]. These gastrointestinal disorders are characterized by a relapsing-remitting disease course with symptoms such as abdominal pain, diarrhea, and rectal bleeding [4,5] and thereby impose not only a physical but also a psychosocial and occupational burden on affected individuals [6]. Therapeutic approaches for IBD vary according to disease stage and symptom severity, but their main aim is to achieve and maintain disease control and to improve quality of life [7,8].

Disease onset commonly occurs between 30 and 40 years of age [9], a period of high labour-market participation and professional development. When the disease begins early in life, and long-term stability is not achieved, it not only places a financial strain on affected individuals but also generates substantial costs for society [10,11]. In addition to the direct costs related to medication, hospital admissions, and surgery, indirect costs due to productivity loss are substantial [12]. Individuals with IBD are more often affected by sickness absence, unemployment, early retirement, and short- or long-term work disability than the general population [13,14]. In Germany, indirect costs represented the second largest cost component for patients with IBD after inpatient costs, ranging from €463 to €3493 per patient per 30 days, according to a systematic literature review [15].

Maintaining work participation is therefore an important objective in the management of IBD [16]. This requires a thorough understanding of the extent of sickness absence and work impairment associated with the disease and its potential impact on productivity and healthcare costs [17]. Previous studies have reported considerable variation in the occurrence and duration of sickness absence and work disability among patients with IBD, which may partly reflect differences in study design, definitions of medical leave, and observation periods [17,18]. Nevertheless, some studies have identified factors associated with work impairment, including higher disease activity, CD compared with UC, absence of medical interventions, female sex, smoking, and anxiety symptoms [14,17,19,20,21,22]. However, findings remain inconsistent for several other factors, such as age, surgical treatment, and educational level [19]. These variations highlight the need for country-specific, population-based studies that quantify the extent of sickness absence associated with IBD in different healthcare settings.

Since evidence on certified sick leave related to IBD in Germany remains scarce, this study aimed to compare the (1) occurrence and (2) duration of certified sick leave, as a routinely recorded proxy for temporary sickness absence, between patients with IBD and matched non-IBD controls before and after diagnosis using real-world data.

## 2. Materials and Methods

### 2.1. Data Source

This retrospective cohort study used data from the Disease Analyzer (DA), a national database (IQVIA) in Germany that comprises anonymized medical practice data. Demographic information (such as age and sex), disease details, and prescription records are available in the database. The data are sourced directly from the electronic medical records of general and specialized practices but are encrypted before transmission to IQVIA for data privacy purposes. Diagnoses and prescriptions within the database are classified according to the International Classification of Diseases, 10th revision (ICD-10), and the Anatomical Therapeutic Chemical (ATC) Classification System of the European Pharmaceutical Marketing Research Association (EphMRA). The DA is deemed representative of the country’s primary care practices [23], and several studies, also on IBD, have previously been conducted using this database [24,25]. No institutional review board approval or informed consent is required under German law for studies using this secondary database.

### 2.2. Study Population

Patients with an initial diagnosis of IBD (ICD-10 K50 for CD or K51 for UC) between 2010 and 2022, and aged 18 years or older at diagnosis, were selected. Additionally, patients had to have an observation time of at least 12 months before and 24 months after the diagnosis date. To increase the likelihood of selecting patients with active employment, individuals covered by family insurance and those aged 65 years or older (as a proxy for retirement) were excluded. Similar criteria applied to the non-IBD controls, with a randomly selected visit date serving as the index date. The controls were matched to the IBD cohort at a ratio of 5:1 using propensity score matching based on age, sex, time period of the index year, insurance status, Charlson comorbidity index, and psychiatric comorbidity (ICD-10 groups F2, F3, and F4) at baseline. A 5:1 matching ratio was chosen to increase the precision of the estimates by making use of the large pool of eligible non-IBD controls while maintaining a practical matching ratio. Propensity score matching was performed once in the overall cohort; in all stratified analyses, each IBD patient was analyzed together with their originally matched controls, so that the balance achieved in the overall matching was preserved without re-matching within strata.

### 2.3. Study Outcomes

A sick leave episode was defined as a single physician-certified period of incapacity for work (Arbeitsunfähigkeit), documented with a start and end date in the electronic medical record; such episodes could last from a single day to several weeks and were not restricted to a minimum number of consecutive days per calendar year. Occurrence of sick leave was defined as the proportion of individuals with at least one registered sick leave episode within each of the three predefined periods relative to the diagnosis (index) year: the pre-index period (12 months before index), the first year post-index (months 1–12), and the second year post-index (months 13–24).

Sick leave duration was calculated as the total number of sick leave days within each annual period, obtained by summing all individual sick leave episodes. In Germany, employees are legally required to provide a sick leave certificate from the fourth day of absence, or earlier, depending on employer regulations.

### 2.4. Statistical Analyses

Baseline characteristics of the cohorts after propensity score matching were described using counts and percentages for categorical variables and means with standard deviations (SDs) or medians with interquartile ranges (IQR) for continuous variables, depending on their distribution. Standardized mean differences (SMDs) were calculated to assess balance in baseline characteristics between the two cohorts, with values of <0.1 indicating negligible differences.

Logistic regression was used to assess the association between IBD and the probability of being on sick leave in each of the three above-mentioned periods. The analysis was also stratified by IBD type, sex, and age groups. Odds ratios (OR) were reported together with 95% confidence intervals (CIs).

Differences in sick leave duration between patients with IBD and matched controls were analyzed using zero-inflated negative binomial (ZINB) regression for each of the three periods. This approach was chosen because the distribution of sick leave days was highly right-skewed with an excess of zero values, indicating that many individuals had no recorded sick leave. Descriptive statistics, therefore, included both medians with IQR and means with SDs. While medians reflect the typical number of sick leave days among individuals, mean values were additionally reported to capture the overall societal burden of sick leave. Stratified analyses by sex and age groups were conducted to explore potential differences in the association between IBD and sick leave across subgroups, while analyses by IBD type compared each subtype with the matched non-IBD controls. Results from the regression analyses are presented as rate ratios (RRs) with 95% CIs.

A two-sided *p*-value < 0.05 was considered statistically significant.

Analyses were carried out using SAS version 9.4 (SAS Institute, Cary, NC, USA).

## 3. Results

### 3.1. Patient Selection

**Figure 1:**
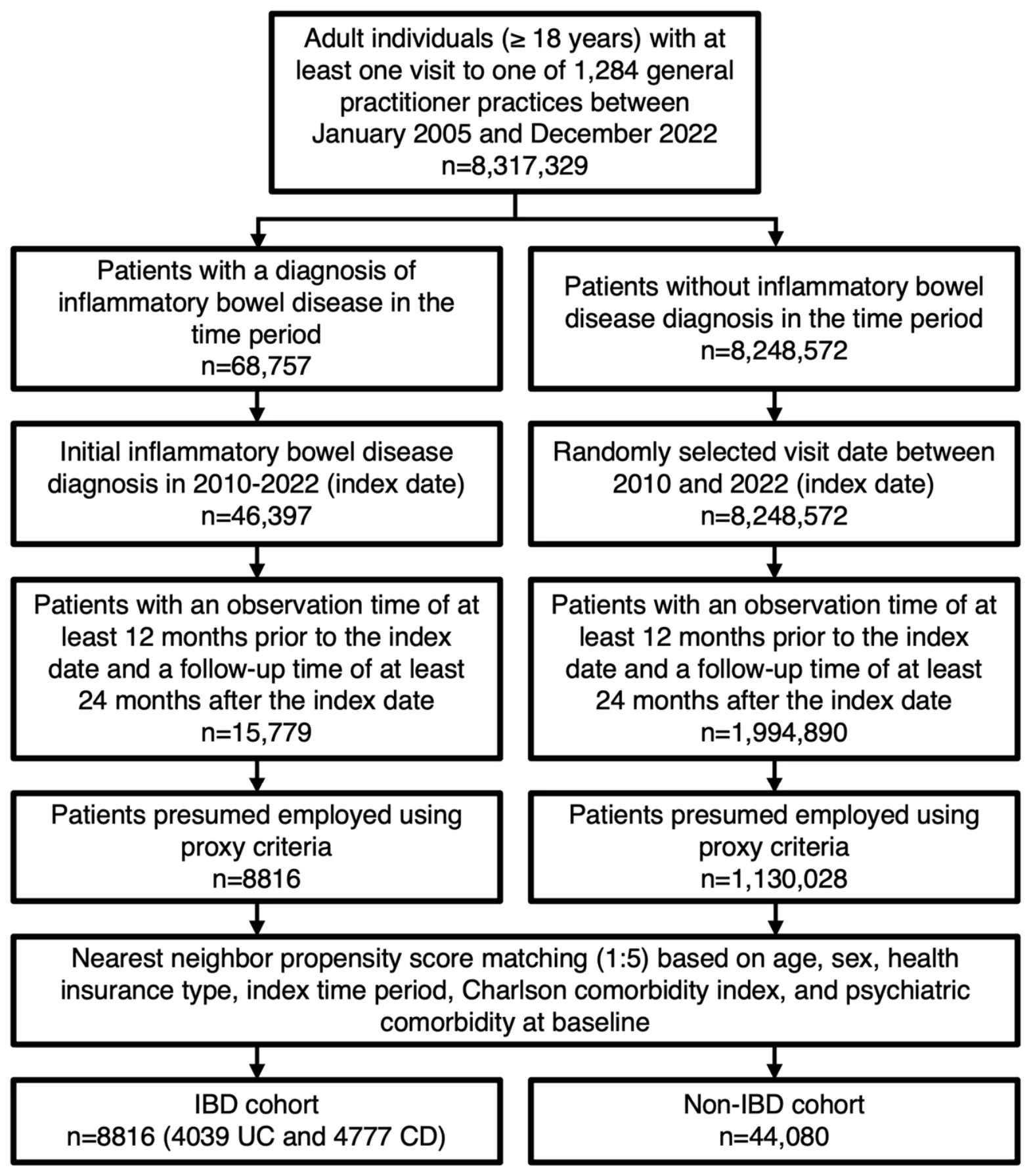
Flowchart of study population selection from the Disease Analyzer database. Adult patients with a newly diagnosed IBD between 2010 and 2022 were identified and screened according to the predefined inclusion and exclusion criteria. Eligible patients were required to have at least 12 months of observation before and 24 months after the index date. Non-IBD controls were selected from the same database and matched to IBD patients at a 5:1 ratio using propensity scores. CD, Crohn’s disease; IBD, inflammatory bowel disease; UC, ulcerative colitis.

A total of 8,317,329 adult patients with at least one practice visit were identified in the DA database between 2005 and 2022, of whom 68,757 had a diagnosis of IBD (Figure 1). Of these, 46,397 were newly diagnosed between 2010 and 2022, and 15,779 had at least 12 months of observation time pre-index and 24 months of follow-up post-index. After applying the proxy criteria for employment, the IBD cohort comprised 8816 patients, of whom 4039 had UC and 4777 had CD.

The non-IBD cohort comprised 44,080 individuals, matched on age, sex, health insurance type, index time period, Charlson comorbidity index, and psychiatric comorbidity at baseline.

### 3.2. Patient Characteristics

After propensity score matching, baseline characteristics were well balanced between the IBD and non-IBD cohorts (Table 1). Both cohorts had a mean (SD) age of 40.2 (12.2) years, with an even distribution across age groups. Approximately half of the patients were female (50.1% in the IBD cohort and 50.0% in the non-IBD cohort), and the majority had statutory health insurance (91.0% and 91.1%, respectively). For most patients, the first visit to the practice occurred between 2019 and 2022 (40.7% in the IBD cohort and 40.5% in the non-IBD cohort), whereas fewer patients had their first visit between 2010 and 2012 (13.0% and 13.1%, respectively). In both cohorts, the median (IQR) Charlson comorbidity index was 0 (1), and psychiatric comorbidities were present in 16.5% of patients.

### 3.3. Patients on Sick Leave

Overall, the proportion of patients with at least one sick leave episode was lowest pre-index and highest in the first year post-index (Table 2). In the IBD cohort, 45.7% of patients had at least one sick leave episode pre-index and 56.2% during the first year post-index, compared with 42.2% and 56.4%, respectively, in the non-IBD cohort. During the second year post-index, the proportion of patients on sick leave decreased to 49.6% in the IBD cohort and to 42.5% in the non-IBD cohort. However, in the IBD cohort, sick leave proportions during the second year post-index remained higher than in the pre-index period, while in the non-IBD cohort, except for individuals aged 18 to 30 years (47.3% pre-index and 45.7% second year post-index), similar proportions were observed in both periods.

Consistent with these descriptive patterns, regression analyses showed that patients with IBD had higher odds of being on sick leave than non-IBD controls pre-index (OR 1.17, 95% CI 1.11–1.22) and during the second year post-index (OR 1.35, 95% CI 1.29–1.42). In contrast, no statistically significant difference between the cohorts was observed during the first year post-index (*p* = 0.764), except for in the stratified analysis among individuals aged 31 to 40 years, where patients with IBD were less likely to be on sick leave than non-IBD controls (OR 0.85, 95% CI 0.77–0.94).

Across stratified analyses, similar patterns were observed for the IBD and non-IBD cohorts. Sick leave was more frequent among patients with UC than among those with CD (e.g., 58.6% vs. 54.2% in the IBD cohort in the first year post-index). Men and women exhibited similar sick leave proportions, except in the pre-index period, where men had a higher proportion (47.8% vs. 43.6% in the IBD cohort). Sick leave proportions were lowest among individuals older than 50 years (e.g., 52.0% vs. 55.2–66.2% in the IBD cohort in the first year post-index) and highest among those aged 18 to 30 years (e.g., 61.2% vs. 52.0–56.3%).

### 3.4. Sick Leave Duration

Median sick leave durations were similar in the IBD and non-IBD cohorts across all time periods (Table 3). The median (IQR) was 0 (0–7) versus 0 (0–5) pre-index, 2 (0–12) versus 2 (0–9) in the first year post-index, and 0 (0–8) versus 0 (0–5) in the second year post-index for the IBD and non-IBD cohorts, respectively. In contrast, mean (SD) sick leave durations were consistently higher in the IBD than in the non-IBD cohort across all periods (6.6 [21.6] vs. 5.4 [20.2] pre-index; 11.4 [31.7] vs. 8.8 [27.2] in the first year post-index; and 9.7 [35.1] vs. 6.1 [24.6] in the second year post-index). This pattern was also reflected by the RRs (95% CI), which were lowest in the year pre-index and highest in the second year post-index. Accordingly, patients with IBD had 1.22 (95% CI 1.15–1.29) and 1.59 (95% CI 1.50–1.69) times more sick leave days than the non-IBD controls in the pre-index period and in the second year post-index, respectively. In both cohorts, the number of sick leave days peaked in the first year post-index based on both mean and median values. Although the median was 0 in both the pre-index period and in the second year post-index, mean sick leave days were lower pre-index and higher in the second year post-index. The differences were primarily driven by the upper end of the distribution, that is, by patients with a high number of sick leave days.

In stratified analyses, patients with UC had slightly higher sick leave durations than those with CD during the post-index periods. In the first year post-index, median (IQR) sick leave days were 3 (0–13) among UC patients and 2 (0–11) among CD patients; during the second year post-index these declined to 1 (0–9) and 0 (0–8), respectively. Non-IBD controls matched to UC and CD patients had identical sick leave durations across all periods, with similar values to the CD patients in the IBD cohort (median [IQR]: 0 [0–5] pre-index, 2 [0–9] in the first year, and 0 [0–5] in the second year post-index). In the IBD cohort, mean (SD) sick leave days were consistently higher for UC than for CD patients in the post-index periods (12.2 [34.0] vs. 10.8 [29.6] in the first year post-index and 10.1 [36.5] vs. 9.3 [33.8] in the second year post-index).

In sex-stratified analyses, men and women had the same median (IQR) number of sick leave days, except for in the first year post-index (3 [0–12] among men vs. 2 [0–11] among women). Mean (SD) sick leave days were similar for both women and men. Across age groups, patients aged 18–30 years showed the highest median sick leave duration in the first year post-index (3 [0–11] days among patients with IBD and 3 [0–8] days among controls). In contrast, the highest mean (SD) number of sick leave days, particularly for this period, was observed among individuals older than 50 years, with 15.5 (44.4) days in the IBD cohort and 10.8 (35.5) days in the non-IBD cohort.

## 4. Discussion

This cohort study demonstrated a higher burden of certified sick leave among patients with IBD than among matched non-IBD controls, particularly during the pre-index period and the second year post-index. Although differences in sick leave proportions and durations between the cohorts were statistically significant in several analyses, the absolute differences were generally modest. Nevertheless, even modest differences may translate into a meaningful societal burden given the chronic nature of IBD and its frequent occurrence during working age. While the proportion of individuals on sick leave peaked during the first year post-index in both cohorts and did not differ significantly between groups during this period, patients with IBD consistently had longer mean sick leave durations. Differences between the cohorts became more apparent during the second year post-index, suggesting a longer-term impact of IBD on work participation beyond the diagnostic period. Stratified analyses further indicated a tendency toward higher sick leave proportions and durations among patients with UC compared with CD and among younger compared with older individuals. However, these subgroup differences were generally small in absolute terms and should therefore be interpreted with caution. In addition, as sick leave episodes could not be linked to their underlying causes, the observed differences should be interpreted as sick leave among patients with IBD rather than sick leave directly attributable to IBD.

### 4.1. Sick Leave Proportions in Patients with IBD

In our IBD study population, sick leave proportions ranged from 45.7% to 56.2% across the observed periods. These estimates are comparable to the pooled sick leave proportion of 39.5% reported in the meta-analysis by Youssef et al. [14] and to the proportions of 47% for UC and 53% for CD patients reported by Bernklev et al. [26]. A multinational study including patients from the Netherlands reported similar but higher sick leave proportions with 59% among patients with UC and 69% among those with CD [27]. In contrast, other studies have reported substantially lower sick leave proportions, ranging from 10% [10] to 21.3% [21]. Comparability across studies is limited by differences in study design, including variation in observation periods (many studies use 12-month periods, whereas Youssef et al. used the Work Productivity and Activity Impairment questionnaire assessing the preceding 7 days), the timing of assessment in relation to diagnosis, and other country-specific differences. In addition, as noted by Youssef et al., disease severity and treatment type may confound sick leave estimates and contribute to variation across studies [14].

### 4.2. Sick Leave Before and After Diagnosis

The temporal pattern observed in our study—characterized by the highest sick leave proportions in the first year post-index, followed by a decline toward pre-index levels—is consistent with findings from Everhov et al. among patients with CD [28]. However, the absolute levels reported by Everhov et al. were substantially lower, with sick leave proportions of 22% pre-index and 34% in the first year post-index, after which they decreased to pre-diagnostic levels. In their comparator population, sick leave proportions remained relatively stable at 8% to 12%. In contrast, sick leave proportions in our non-IBD controls ranged from 42.2% to 56.4%. The discrepancies between the studies are likely attributable to differences in both the assessment of sick leave and the selection of comparator populations. Compared with our study, Everhov et al. excluded sick leave episodes shorter than two weeks and compared patients with the general population [28], whereas our study captured all legally (≥4 days) or employer-required sick leave days and used matched controls selected from routine care.

### 4.3. Patterns of Sick Leave Duration

Median values were similarly low in both cohorts and indicate that most individuals had few or no sick leave days. Youssef et al. also found that absenteeism (16% in IBD vs. 1.5–3% in the non-IBD population) was less affected by IBD than presenteeism (50% vs. 23%, respectively) [14], possibly due to patients fearing financial or job loss [29]. The disease may therefore primarily affect work productivity rather than sick leave absence [14,30], or lead to absence levels that remain below reporting thresholds and are therefore not captured in routine data. Workplace accommodations may be necessary to improve employment outcomes of IBD patients, although many patients face difficulties requesting these from employers [14,31]. The higher mean number of sick leave days among IBD patients compared with non-IBD controls suggests that a small group of patients experienced long sick leave periods. Similar trends were reported by Everhov et al. for patients with CD [28], despite the mean sick leave days in their IBD cohort being higher, at 16, 31, and 21 days in the years before, during, and after diagnosis, respectively. Their control group showed levels comparable to ours, with mean values of 7, 6, and 5 sick leave days. This study, however, did not take sick leave periods of less than 2 weeks that were compensated by the employer into account [28]. While direct comparison with the study by Bernklev et al. is limited by their use of a five-year observation period post diagnosis, that study likewise found that a relatively small proportion of patients (25%) accounted for a large share of total sick leave days [26]. From a public health perspective, this implies that interventions may need to target high-risk subgroups rather than the entire IBD population.

### 4.4. Potential Explanations for Observed Differences

The higher odds of sick leave for the IBD cohort before diagnosis may reflect the burden of undiagnosed disease [28], including symptoms such as abdominal pain, fatigue, and diarrhea [5] that impair work ability. The finding supports the existing literature on diagnostic delay in IBD [28,32] and is consistent with the hypothesis that earlier diagnosis might reduce pre-diagnostic sickness absence, though this warrants further investigation. The peak in sick leave during the first year post-index may be associated with increased healthcare utilization and short-term disease instability. The observation of a similar peak in the non-IBD cohort suggests that any acute health event, rather than IBD alone, may temporarily increase the likelihood of sick leave. Accordingly, the first year post-index may be considered a transitional period, whereas the second year post-index may better reflect the longer-term impact of IBD on work participation. A similar downward trend in sick leave after the first year post-index was also reported by Everhov et al., who suggested that this pattern may reflect partial recovery after treatment initiation [28]. The more apparent differences in sick leave between the cohorts in the second year post-index may reflect the chronic disease burden of IBD, potentially including flares and treatment-related side effects, though this cannot be confirmed from the available data. At the same time, the generally modest differences observed between the cohorts may indicate that many patients adapt to living and working with IBD over time. The first year post-index may therefore represent an important period during which workplace accommodations such as flexible working hours, remote work options, and additional breaks could help patients maintain work participation [33].

### 4.5. Differences Across Patient Subgroups

In the stratified analyses, a higher proportion of patients with UC than with CD were on sick leave and for longer periods, which was descriptively different from findings reported elsewhere [14,19]. Previous evidence has suggested poorer work outcomes among CD patients, possibly reflecting a greater systemic and psychological disease burden than in patients with UC [14]. Likewise, CD was found to possibly predict work disability due to its more severe and progressive disease course. However, the same review also indicated that UC may be more strongly associated with temporary work disability, while CD may be linked to more permanent disability, possibly due to greater structural damage over time [19]. Given that our analyses did not directly compare UC and CD, the observed differences should be interpreted descriptively. One possible explanation for differences between studies could be unmeasured confounding, such as differences in disease course, symptom burden, or treatment patterns [19]. Alternatively, the observed discrepancies may reflect variations in healthcare utilization [34], which could influence sick leave certification and duration.

Sex-related differences were less pronounced in our study, with the exception of the pre-index period, during which men exhibited a higher proportion of sick leave. Although previous research has more frequently identified women as being affected by work disability, findings remain inconsistent. Differences in disease manifestations, occupational factors, and social roles may contribute to the observed variation between men and women [19].

Age-related differences in sick leave were observed in our study, with younger individuals showing higher sick leave proportions and patients older than 50 years exhibiting longer mean sick leave durations during the first year post-index. Evidence regarding the influence of age on work disability in IBD remains inconsistent [19]. The higher sick leave proportion among younger individuals may reflect differences in health-seeking behaviour or sick leave certification patterns across age groups [35], while the higher mean duration among older patients, likely driven by a small subgroup with particularly long sick leave episodes, could be related to other physical health issues [36].

### 4.6. Strengths and Limitations

This study is based on a large number of patients, increasing the representativeness of the findings for the working-age IBD population in Germany. A further strength is the use of matched non-IBD controls, which helps reduce confounding and allows for a more robust comparison of sick leave patterns. In addition, the longitudinal study design enabled the assessment of sick leave both in the pre- and post-index periods, providing insight into temporal changes around diagnosis. Another strength is the assessment of both sick leave occurrence and duration, providing a more comprehensive picture of sickness absence than either measure alone. To our knowledge, this is also the first study in Germany to analyze sick leave using routinely collected healthcare data among IBD patients.

Several limitations should be considered. The burden of sick leave may have been underestimated, as short-term absences of fewer than four days that did not require medical certification were not captured. In addition, because the comparator group did not represent a healthy population, differences between groups may underestimate the true excess burden of IBD relative to the general working population. The control index date reflected a random healthcare contact rather than a first diagnosis, as in the IBD cohort, which may also have influenced healthcare utilization and sick-leave patterns around the index date. Further, the employment proxy used in this study (exclusion of individuals on family insurance and those aged ≥65 years) could not distinguish unemployed individuals, the self-employed, part-time workers, or those receiving long-term disability benefits. Consequently, the study population represents a selected subset of working-age individuals, limiting the generalizability of the findings. Moreover, the retrospective cohort design may have limited the completeness and consistency of routinely recorded sick-leave data and the availability of potential confounding factors like employment characteristics (e.g., occupation, working conditions, industry, company size), socioeconomic status, lifestyle factors, and disease severity or activity (e.g., endoscopic data). Such detailed employment information is not routinely captured in the Disease Analyzer database and, because the data are anonymized, cannot be linked to external employment or social-security records; obtaining it would require prospective data collection. Where available, characteristics such as occupation, industry, and company size could refine the estimates, given their known association with sick leave patterns. Although prescription records were available, treatment information (e.g., biologic therapy, immunosuppressants, and surgical interventions) was not included as a covariate because patients’ treatment exposure varied over time and patients could receive multiple treatment modalities. As these factors may substantially influence work capacity and sick leave patterns [14,19], residual confounding cannot be excluded. Also, the database does not link sick leave episodes to their underlying cause. It is therefore unknown whether sick leave in IBD patients is attributable to IBD itself, to comorbidities, or to unrelated conditions, which substantially limits causal inference. Additionally, as diagnoses were based on routinely recorded ICD-10 codes, some degree of diagnostic misclassification cannot be ruled out. Sick leave episodes were only captured when patients consulted their GP to obtain a certificate; thus, episodes among patients managed outside primary care or exclusively by specialists may have been missed, potentially underestimating the true burden of sick leave. In addition, the study period (2010–2022) spans a time during which ICD coding practices and data completeness in the Disease Analyzer database may have changed, introducing potential measurement variability. Finally, the COVID-19 pandemic period (2020–2022) may also have influenced sick leave patterns in the most recent years of the observation period; this potential temporal confounding was not specifically examined.

## 5. Conclusions

This study demonstrated a modest but persistent excess burden of certified sick leave among patients with IBD compared with matched non-IBD controls. These findings support considering work participation and sick leave alongside other patient-relevant outcomes, such as disease activity and remission, in the management of IBD. The occupational burden of IBD may begin before diagnosis and persist beyond the initial diagnostic period, with differences between patients with IBD and controls becoming most apparent in the second year after diagnosis. These findings suggest that work-related difficulties should be considered early in the course of IBD and monitored beyond the diagnostic period. Early identification of patients experiencing work impairment may facilitate timely disease management and workplace support, including flexible working hours, remote work, and additional breaks [33], particularly during periods of active disease or treatment changes. Given that the higher mean sick leave duration occurred despite relatively similar proportions and low median values, support may be particularly relevant for a subgroup of patients experiencing prolonged or recurrent sick leave rather than the IBD population as a whole.

Future research should evaluate whether such targeted approaches can improve sustained work participation and reduce prolonged sickness absence. Studies should move beyond certified sick leave to capture the broader occupational impact of IBD, including presenteeism, reduced work productivity, working hours, employment status, and uncertified short-term absences. As suggested by previous research [14], the impact of IBD on presenteeism may exceed its effect on sick leave or absenteeism, indicating that the disease’s full occupational burden may not be captured by sick leave alone. Future studies should also identify patients at higher risk of prolonged work absence and examine the contribution of, e.g., disease activity, treatment, comorbidities, occupational characteristics, and workplace accommodations. This could help identify modifiable factors and inform targeted interventions aimed at maintaining work participation and preventing avoidable loss of employment among patients with IBD.

## Figures and Tables

**Table 1:** Baseline characteristics of study patients after 1:5 matching.

Characteristic	Category	IBD Cohort N (%)	Non-IBD Cohort N (%)	SMD
Total		8816 (100%)	44,080 (100%)	
Age (years)	Mean (SD)	40.2 (12.2)	40.2 (12.2)	0.000
	18–30	2331 (26.4)	11,639 (26.4)	0.000
	31–40	2067 (23.5)	10,348 (23.5)	
	41–50	2198 (24.9)	11,007 (25.0)	
	>50	2220 (25.2)	11,086 (25.1)	
Sex	Female	4416 (50.1)	22,056 (50.0)	0.001
	Male	4400 (49.9)	22,024 (50.0)	
Health insurance	Statutory	8024 (91.0)	40,157 (91.1)	−0.001
	Private	792 (9.0)	3923 (8.9)	
Year of inclusion	2010–2012	1150 (13.0)	5786 (13.1)	0.003
	2013–2015	1790 (20.3)	8959 (20.3)	
	2016–2018	2295 (26.0)	11,481 (26.1)	
	2019–2022	3581 (40.7)	17,854 (40.5)	
Charlson comorbidity index	Median (IQR)	0 (1)	0 (1)	0.022
Psychiatric comorbidity	Yes	1456 (16.5)	7265 (16.5)	0.000
	No	7360 (83.5)	36,815 (83.5)	

Data are absolute numbers and percentages, unless otherwise specified. SD, standard deviation; SMD, standardized mean difference; IQR, interquartile range.

**Table 2:** Comparison of the number of patients on sick leave between the IBD and non-IBD cohorts in each period, overall and by stratum.

Strata	Period	IBD Cohort (%)	Non-IBD Cohort (%)	Odds Ratio (95% CI) ^a^	*p*-Value ^a^
Total	Pre-index	45.7	42.2	1.17 (1.11–1.22)	<0.001
	First year post-index	56.2	56.4	0.99 (0.95–1.04)	0.764
	Second year post-index	49.6	42.5	1.35 (1.29–1.42)	<0.001
Crohn’s disease	Pre-index	45.1	41.3	1.18 (1.11–1.26)	<0.001
	First year post-index	54.2	55.7	0.94 (0.88–1.00)	0.050
	Second year post-index	48.2	41.9	1.31 (1.23–1.40)	<0.001
Ulcerative colitis	Pre-index	46.4	43.3	1.15 (1.07–1.23)	<0.001
	First year post-index	58.6	57.1	1.06 (0.99–1.14)	0.091
	Second year post-index	51.1	43.1	1.41 (1.31–1.51)	<0.001
Women	Pre-index	43.6	41.8	1.08 (1.01–1.16)	0.019
	First year post-index	55.8	55.1	1.03 (0.97–1.10)	0.347
	Second year post-index	49.5	42.2	1.36 (1.28–1.46)	<0.001
Men	Pre-index	47.8	42.6	1.26 (1.17–1.34)	<0.001
	First year post-index	56.7	57.7	0.96 (0.89–1.02)	0.180
	Second year post-index	49.6	42.7	1.35 (1.26–1.44)	<0.001
Age 18–30 years	Pre-index	48.3	47.3	1.04 (0.95–1.14)	0.398
	First year post-index	61.2	63.3	0.91 (0.83–1.00)	0.053
	Second year post-index	52.3	45.7	1.31 (1.20–1.44)	<0.001
Age 31–40 years	Pre-index	46.8	44.3	1.11 (1.01–1.23)	0.033
	First year post-index	56.3	60.2	0.85 (0.77–0.94)	0.001
	Second year post-index	50.9	44.9	1.29 (1.17–1.42)	<0.001
Age 41–50 years	Pre-index	45.0	39.6	1.28 (1.16–1.41)	<0.001
	First year post-index	55.2	53.4	1.08 (0.99–1.19)	0.094
	Second year post-index	48.9	40.9	1.42 (1.29–1.56)	<0.001
Age > 50 years	Pre-index	42.7	37.4	1.26 (1.15–1.39)	<0.001
	First year post-index	52.0	48.7	1.15 (1.05–1.27)	0.004
	Second year post-index	46.1	38.5	1.41 (1.28–1.55)	<0.001

^a^ Conditional logistic regression.

**Table 3:** Comparison of the sick leave days between the IBD and non-IBD cohorts in each period, overall and by stratum.

Strata	Period	IBD Median (IQR)	Non-IBD Median (IQR)	IBD Mean (SD)	Non-IBD Mean (SD)	Rate Ratio (95% CI) ^a^	*p*-Value ^a^
Total	Pre-index	0 (0–7)	0 (0–5)	6.6 (21.6)	5.4 (20.2)	1.22 (1.15–1.29)	<0.001
	First year post-index	2 (0–12)	2 (0–9)	11.4 (31.7)	8.8 (27.2)	1.30 (1.24–1.36)	<0.001
	Second year post-index	0 (0–8)	0 (0–5)	9.7 (35.1)	6.1 (24.6)	1.59 (1.50–1.69)	<0.001
Crohn’s disease	Pre-index	0 (0–7)	0 (0–5)	6.6 (20.7)	5.3 (20.4)	1.24 (1.14–1.34)	<0.001
	First year post-index	2 (0–11)	2 (0–9)	10.8 (29.6)	8.8 (27.9)	1.22 (1.14–1.30)	<0.001
	Second year post-index	0 (0–8)	0 (0–5)	9.3 (33.8)	6.2 (26.1)	1.50 (1.38–1.62)	<0.001
Ulcerative colitis	Pre-index	0 (0–7)	0 (0–5)	6.6 (22.6)	5.5 (20.0)	1.19 (1.10–1.30)	<0.001
	First year post-index	3 (0–13)	2 (0–9)	12.2 (34.0)	8.8 (26.4)	1.39 (1.29–1.49)	<0.001
	Second year post-index	1 (0–9)	0 (0–5)	10.1 (36.5)	5.9 (22.7)	1.71 (1.59–1.86)	<0.001
Women	Pre-index	0 (0–6)	0 (0–5)	6.3 (22.3)	5.3 (20.4)	1.18 (1.09–1.29)	<0.001
	First year post-index	2 (0–11)	2 (0–8)	11.1 (32.0)	8.4 (26.2)	1.32 (1.23–1.41)	<0.001
	Second year post-index	0 (0–8)	0 (0–5)	9.7 (35.3)	6.0 (24.4)	1.62 (1.49–1.76)	<0.001
Men	Pre-index	0 (0–7)	0 (0–5)	6.9 (20.9)	5.5 (20.1)	1.25 (1.15–1.35)	<0.001
	First year post-index	3 (0–12)	2 (0–9)	11.7 (31.4)	9.2 (28.2)	1.28 (1.19–1.36)	<0.001
	Second year post-index	0 (0–8)	0 (0–5)	9.7 (34.8)	6.2 (24.9)	1.57 (1.44–1.70)	<0.001
Age 18–30 years	Pre-index	0 (0–6)	0 (0–5)	5.3 (14.9)	4.3 (11.6)	1.23 (1.11–1.35)	<0.001
	First year post-index	3 (0–11)	3 (0–8)	9.6 (23.7)	7.0 (17.0)	1.37 (1.27–1.48)	<0.001
	Second year post-index	1 (0–8)	0 (0–5)	7.4 (23.7)	4.7 (15.9)	1.59 (1.43–1.75)	<0.001
Age 31–40 years	Pre-index	0 (0–7)	0 (0–5)	5.9 (16.7)	5.0 (17.1)	1.19 (1.07–1.34)	0.002
	First year post-index	2 (0–11)	3 (0–10)	9.5 (23.2)	8.1 (21.0)	1.16 (1.06–1.28)	0.001
	Second year post-index	1 (0–8)	0 (0–6)	8.8 (32.8)	5.3 (19.3)	1.65 (1.47–1.84)	<0.001
Age 41–50 years	Pre-index	0 (0–7)	0 (0–5)	6.5 (20.0)	5.5 (21.6)	1.18 (1.04–1.33)	0.008
	First year post-index	2 (0–12)	2 (0–8)	11.1 (30.2)	9.4 (31.1)	1.19 (1.07–1.31)	0.001
	Second year post-index	0 (0–8)	0 (0–5)	10.1 (36.9)	6.3 (26.5)	1.60 (1.41–1.80)	<0.001
Age >50 years	Pre-index	0 (0–8)	0 (0–5)	8.6 (31.0)	6.9 (27.3)	1.26 (1.10–1.44)	0.001
	First year post-index	2 (0–13)	0 (0–9)	15.5 (44.4)	10.8 (35.5)	1.43 (1.28–1.60)	<0.001
	Second year post-index	0 (0–10)	0 (0–6)	8.1 (33.1)	6.9 (27.3)	1.56 (1.37–1.78)	<0.001

^a^ Zero-inflated negative binomial (ZINB) regression. IQR, interquartile range; SD, standard deviation.

## Data Availability

The data and the code used for this study are available from the corresponding author upon reasonable request.

## Abbreviations

The following abbreviations are used in this manuscript:

IBD	Inflammatory bowel disease
CD	Crohn’s disease
UC	Ulcerative colitis
DA	Disease Analyzer
ICD-10	International Classification of Diseases, 10th revision
ATC	Anatomical Therapeutic Chemical
OR	Odds ratio
RR	Rate ratio
CI	Confidence interval
SD	Standard deviation
IQR	Interquartile range
SMD	Standardized mean difference
ZINB	Zero-inflated negative binomial
